# Survival Differences Between Individuals With Typical and Atypical Phenotypes of Alzheimer Disease

**DOI:** 10.1212/WNL.0000000000213603

**Published:** 2025-04-28

**Authors:** Ilse Bader, Colin Groot, Wiesje M. Van Der Flier, Yolande A.L. Pijnenburg, Rik Ossenkoppele

**Affiliations:** 1 Amsterdam Neuroscience, Neurodegeneration, the Netherlands;; 2 Alzheimer Center Amsterdam, Neurology, Vrije Universiteit Amsterdam, Amsterdam UMC Location VUmc, the Netherlands;; 3 Epidemiology and Data Science, Vrije Universiteit Amsterdam, Amsterdam UMC Location VUmc, the Netherlands; and; 4 Clinical Memory Research Unit, Lund University, Lund, Sweden.

## Abstract

**Background and Objectives:**

Survival estimates for individuals with Alzheimer disease (AD) are informative to understand the disease trajectory, but precise estimates for atypical AD variants are scarce. Atypical AD variants are characterized by nonamnestic phenotypes, an early onset, and lower prevalence of *APOE*ε*4* carriership, which affect the AD trajectory. We aimed to provide survival estimates for posterior cortical atrophy (PCA), logopenic variant primary progressive aphasia (lvPPA), and behavioral AD (bvAD) and to evaluate the effect of these atypical AD diagnoses beyond known mortality determinants.

**Methods:**

From the Amsterdam Dementia Cohort, we retrospectively selected patients with biomarker-confirmed sporadic AD presenting at the memory clinic in the mild cognitive impairment or dementia stage. Patients were classified into atypical AD phenotypes (PCA, lvPPA, bvAD; multidisciplinary consensus and retrospective case finding) and a typical AD reference group (excluding unclassifiable atypical presentations or unconfirmed future AD dementia). Survival estimates from the first visit to death/censoring (Central Public Administration) were determined (Kaplan-Meier analysis) and compared (log-rank tests) across diagnostic groups. To assess associations of atypical AD with mortality, Cox proportional hazard models were constructed including age, sex, education, MMSE score, and *APOE*ε*4* carriership (model 1), followed by adding the atypical AD group (model 2) or atypical AD variants (model 3). A likelihood ratio test was performed to compare the fit of model 1 and model 2.

**Results:**

A total of 2,081 patients (aged 65 ± 8 years, 52% female) were classified as typical AD (n = 1,801) or atypical AD (n = 280; PCA [n = 112], lvPPA [n = 86], and bvAD [n = 82]). The estimated median survival time for atypical AD of 6.3 years (95% CI [5.8–6.9]) was shorter than for typical AD (7.2 [7.0–7.5], *p* = 0.02). Median survival durations across the atypical AD variants were consistently, albeit nonsignificantly, shorter (PCA: 6.3 [5.5–7.3], *p* = 0.055; lvPPA: 6.6 [5.7–7.7], *p* = 0.110; bvAD: 6.3 [5.0–9.1], *p* = 0.121, 48% deceased). Including atypical AD improved the model fit (model 2; χ^2^ = 8.88, *p* = 0.003) and was associated with 31% increased mortality risk compared with typical AD (hazard ratio [HR] = 1.31 [1.10–1.56], *p* = 0.002). In model 3, contributions of the variants were as follows: HR_PCA_ = 1.35 (1.05–1.73), *p* = 0.019; HR_lvPPA_ = 1.27 (0.94–1.69), *p* = 0.114; HR_bvAD_ = 1.31 (0.94–1.83), *p* = 0.105.

**Discussion:**

Survival in atypical AD (PCA, lvPPA, bvAD) was shorter compared with typical AD. These atypical variants are associated with increased mortality beyond age, sex, education, *APOE*ε*4* carriership, and disease severity. Future studies are required to address generalizability of these findings and to identify factors that explain the observed survival differences.

## Introduction

Alzheimer disease (AD) is characterized by presence of amyloid β (Aβ) plaques and tau neurofibrillary tangles that lead to a gradual decline in cognitive function, typically starting with predominant memory disturbances. Atypical clinical variants of AD are characterized by various nonamnestic phenotypes, including predominant disturbances in processing of visual information in posterior cortical atrophy (PCA),^1^ language deficits in logopenic variant primary progressive aphasia (lvPPA),^2^ and behavioral and personality changes in behavioral AD (bvAD).^3^ Apart from a nonamnestic phenotype, atypical AD variants are associated with a relatively young age at onset (65 years or younger) and a lower prevalence of *APOE*ε*4* carriership.^4^ These key characteristics are known modulators of the AD clinical trajectory^5-12^ and determinants of mortality risk,^5,9,13-15^ already suggesting that clinical trajectories may differ in typical vs atypical AD. As comparisons between the clinical trajectories of typical AD vs atypical AD variants are often complicated because of their distinct cognitive and pathophysiologic trajectories, we consider survival as a relatively generalizable outcome to further evaluate whether these atypical AD diagnoses are related to mortality above and beyond known risk determinants.

Expected survival in AD cohorts is shorter compared with the general population, but specific estimates vary (e.g., mean survival of 5.8 or 6.3 years from diagnosis and 6.9 or 7.6 years from disease onset^16,17^) and are known to be affected by factors including age, disease stage,^18,19^ sex,^20,21^ education (largely attributable to differential exposure to lifestyle risk factors^22,23^), and genetic factors such as *APOE*ε*4* carriership.^13^ Because these survival estimates are obtained from mostly older and typical AD samples, they are not directly translatable to patients with atypical AD. After all, atypical AD variants are characterized by predominant nonamnestic phenotypes and a young disease onset (both related to faster disease progression^5-9,14,24-26^) and by a lower prevalence of *APOE*ε*4* carriership (known for differential pleiotropic associations across the AD trajectory^10,11^ and on mortality risk^13^). Furthermore, specific estimates of survival in atypical variants of AD thus far have been variable (e.g., median survival of 8 years from study enrollment^27^ or 10.3 years from symptom onset^28^ for PCA), scarce (e.g., a single study reported a median survival of 6 years from symptom onset and 5 years from diagnosis for lvPPA^29^), or yet undetermined (i.e., for bvAD). Furthermore, although lvPPA^30^ and PCA^31^ are predominantly related to AD pathology, these previous estimates are obtained from samples that lack AD biomarker verification.^27-29^

Taken together, survival estimates for typical AD may not apply to atypical AD, and survival estimates specific to atypical AD are scarce. Furthermore, it remains unclear whether atypical AD diagnoses are related to mortality above and beyond known determinants of mortality. Gaining insights into factors contributing to mortality risk in atypical AD is important to grasp the full disease trajectory and to further identify relevant prognostic factors. Therefore, we aimed (1) to provide survival estimates for biomarker-confirmed typical AD and atypical AD variants (PCA, lvPPA, and bvAD) and (2) to evaluate the effect of these atypical AD diagnoses while taking into account other determinants (sex, education, disease stage, *APOE*ε*4* carriership, and age at onset).

## Methods

### Participants

Patients were selected from the Amsterdam Dementia Cohort (ADC), a memory clinic–based cohort that includes data collected as part of a standard diagnostic workup and clinical follow-up.^32^ The ADC does not apply formal exclusion criteria and includes a heterogeneous cohort of patients who presented at the Alzheimer Center Amsterdam and provided informed consent for their clinical data to be used in research. The standardized diagnostic workup entails a standard dementia screening including medical, neurologic, physical, and neuropsychological investigation; informant-based history taking; brain imaging; standard laboratory results; and biomarker assessment based on CSF and/or PET.^32^ A subsequent diagnosis of dementia or mild cognitive impairment (MCI) due to AD is made during a multidisciplinary consensus meeting in compliance with core clinical criteria of the National Institute on Aging–Alzheimer's Association criteria.^33,34^ For this study, the term atypical AD specifically refers to a PCA, lvPPA, or bvAD diagnosis, and this definition should be taken into account when interpreting the results. These diagnoses are based on multidisciplinary consensus diagnosis and/or on retrospective case finding. Retrospective case finding was specifically required for patients who had received a clinical diagnosis before the criteria for atypical AD phenotypes became available.^4,35^ Implemented criteria were PCA criteria of Crutch et al. (2017),^36^ Mendez et al. (2002),^37^ or Tang-Wai et al. (2004)^38^; lvPPA criteria of Gorno-Tempini et al. (2011)^2^; and bvAD criteria of Ossenkoppele et al.^3^ (2022).

**Figure 1:**
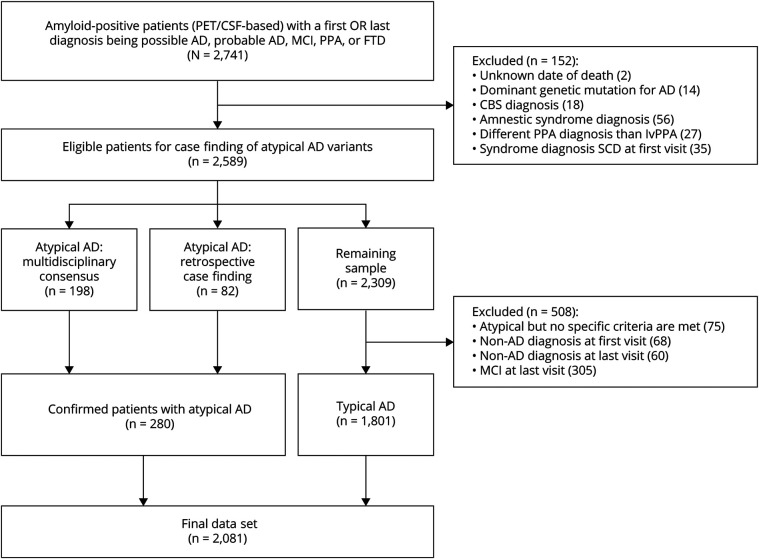
Flow Diagram of the Study Sample AD = Alzheimer disease; bvAD = behavioral variant AD; CBS = corticobasal syndrome; lvPPA = logopenic variant primary progressive aphasia; MCI = mild cognitive impairment; PCA = posterior cortical atrophy; SCD = subjective cognitive decline.

For this study, patients from the ADC were included or excluded in accordance with the flow diagram shown in Figure 1. Inclusion criteria were (1) amyloid positivity based on visual read of Aβ-PET or CSF analysis and (2) possible AD, probable AD, MCI, PPA (i.e., including possible lvPPA), or frontotemporal dementia (FTD, i.e., potentially including bvAD) as first or last diagnosis. Exclusion criteria were (1) an unknown date of death, (2) a dominant genetic variant leading to AD, (3) a corticobasal syndrome (CBS) diagnosis, (4) an amnestic syndrome diagnosis, (5) a different PPA diagnosis than lvPPA, and (6) a syndromic diagnosis of subjective cognitive decline (SCD) at the first visit. In summary, we focus on patients with biomarker-confirmed sporadic AD who presented at the memory clinic in the MCI or dementia stage. In this population, Aβ-PET was performed when a lumbar puncture was contraindicated and/or in context of research for 417 individuals. Aβ-PET was performed using the 11C-Pittsburgh compound B, 18F-flutemetamol, and 18F-florbetaben tracers, followed by visual assessment by trained readers. CSF was available for 1,956 individuals. Before 2018, the drift-corrected Aβ42 concentration was used (Innotest, Fujirebio, Gent, Belgium) with a cutoff of <813 pg/mL for amyloid positivity (n = 1,402). After 2018, the Elecsys Aβ42 concentration was used (Roche, Rotkreuz, Switzerland) with a cutoff of <1,000 pg/mL indicating amyloid positivity (n = 552). For 294 individuals, both PET and CSF were available and discordance was observed in only 21 individuals (1%; 13 PET-positive/CSF-negative, 8 PET-negative/CSF-positive).

Within this sample, patients were classified into atypical and typical AD diagnostic groups. Atypical patients with AD were (1) identified during multidisciplinary consensus meetings and/or previous research studies^30,39-41^ or (2) identified based on case finding after detailed retrospective inspection of patient files by IB, YP, and RO in cases where the differential diagnosis or clinical presentation indicated a potential atypical presentation based on comments in the patient charts mentioning atypicality or a nonamnestic phenotype of interest. The typical AD diagnostic group was further specified by excluding (1) patients who were identified as atypical during retrospective case finding but did not meet all criteria for a specific variant, (2) patients with a non-AD diagnosis at the first visit or at the last visit, and (3) patients with an MCI diagnosis at the *last* visit. In summary, the typical AD group does not include patients with an atypical presentation or without confirmed presence of future AD dementia.

### Data Collection

Survival was defined as the time from the first diagnostic workup to the event (being either death or censoring). Mortality data were collected from the Central Public Administration in the Netherlands until June 2024 and included the status (dead/alive) and date of death. If data from the Central Public Administration were not available, the date of the last visit was included as censoring date. Patients were censored at the known last date of being alive or in case of known euthanasia (n = 13; PCA [n = 2], bvAD [n = 1]), typical AD [n = 10]). The date of the first visit was preferred over the time of diagnosis to avoid any bias due to a diagnostic delay, although the interval between the time of the first visit and time of diagnosis was negligible (0.12 ± 0.73 months on average) and did not differ across the diagnostic groups (H(3) = 4.30, *p* = 0.231).

Age, sex, and education were collected at the first visit. Education was operationalized using the ordinal 7-item Dutch Verhage scale ranging from <6 years of elementary school (score = 1) to a university degree (score = 7). Concern duration is the self-reported duration of concerns at the first visit in years. The MMSE score was obtained as part of neuropsychological testing at or around the first visit. *APOE* genotyping was based on DNA isolated from EDTA blood samples and performed at the Department of Clinical Chemistry of the VUmc using the LightCycler *APOE* variant detection method (Roche Diagnostics GmbH, Mannheim, Germany). The resulting genotype was subsequently grouped into *APOE*ε*4* noncarriers (*APOE*ε2/ε2 [n = 2], *APOE*ε2/ε3 [n = 61], *APOE*ε3/ε3 [n = 538]), heterozygote carriers (*APOE*ε2/ε4 [n = 39], *APOE*ε3/ε4 [n = 875]), and homozygote carriers (*APOE*ε*4*/ε4 [n = 457]).

Cognitive domain scores were determined using neuropsychological assessments performed within 1 year from the first visit. Five domains were evaluated: memory (Visual Association Test [VAT], Rey Auditory Verbal Learning Test immediate and delayed recall), executive functioning (digit-span backward, Trail Making Test [TMT] part B, Stroop test form III, letter fluency), visual-spatial functioning (number location, dot counting, and fragmented letters of the Visual Object and Space Perception Battery), language (category fluency, VAT naming), and attention (TMT-A, Stroop I/II, digit-span forward). TMT and Stroop scores were inverted and log-transformed. Test scores were converted to *Z*-scores based on the study sample's mean and SD and were combined and averaged within each domain to indicate group performance relative to the full sample (Table).

**Table:** Demographic and Clinical Characteristics

	Typical AD (A) N = 1,802	PCA (B) N = 112	lvPPA (C) N = 86	bvAD (D) N = 82	*p* Value
Age at first visit (y)	66 (8)^B^	61 (6)^A,C,D^	68 (7)^B^	65 (8)^B^	<0.001
Deceased, n (%)	1,137 (63)^D^	74 (66)	54 (63)	39 (48)^A^	0.033
Age at death (y)	73 (8)^B^	67 (5)^A,C^	74 (7)^B^	70 (9)	<0.001
Follow-up time (y)	5.5 (3.5)^D^	5.2 (2.6)	5.0 (2.8)	4.1 (2.7)^A^	0.002
Female, n (%)	940 (52)^B,D^	75 (67)^A,C,D^	37 (43)^B^	25 (30)^A,B^	<0.001
Education level (Verhage)^a^	5 (4, 6)	5 (4, 6)	5 (4, 6)	5 (4, 6)	0.8
APOEε4 carriership					<0.001
Noncarrier, n (%)	476 (28)^B,C^	49 (46)^A^	48 (56)^A,D^	28 (35)^C^	<0.001
Heterozygote, n (%)	803 (47)^C^	47 (44)	27 (32)^A,D^	37 (46)^C^	0.045
Homozygote, n (%)	421 (25)^B,C^	11 (10)^A^	10 (12)^A^	15 (19)	<0.001
Syndrome diagnosis					<0.001
Dementia, n (%)	1,566 (87)^B^	112 (100)^A,D^	82 (95)	75 (91)^B^	
MCI, n (%)	235 (13)^B^	0 (0)^A,D^	4 (4.7)	7 (8.5)^B^	
CDR score					0.018
≤0.5, n (%)	655 (43)^B^	29 (29)^A^	43 (51)	32 (43)	
≥1.0, n (%)	874 (57)^B^	71 (71)^A^	41 (49)	43 (57)	
MMSE (total score)	21.2 (5.3)	20.3 (4.5)	21.4 (5.7)	22.4 (4.6)	0.061
Concern duration (y)^a^	3 (2, 4)	3 (2, 4)	2.50 (2, 4)	3 (2, 5)	0.011
Age at diagnosis					<0.001
Older than 65 years, n (%)	919 (51)^B,C^	23 (21)^A,C,D^	60 (70)^A,B,D^	41 (50)^B,C^	
65 years or younger, n (%)	882 (49)^B,C^	89 (79)^A,C,D^	26 (30)^A,B,D^	41 (50)^B,C^	
Cognitive domain scores					
Memory	−0.03 (0.75)^B,C,D^	0.35 (1.07)^A^	0.64 (1.07)^A^	0.44 (0.85)^A^	<0.001
Executive function	0.19 (0.75)^B,C^	−0.15 (0.79)^A^	−0.15 (0.62)^A^	0.04 (0.75)	<0.001
Language	0.06 (0.79)^B,C^	−0.17 (0.90)^A,C,D^	−0.55 (1.09)^A,B,D^	0.16 (0.77)^B,C^	<0.001
Visuospatial	0.12 (0.60)^B^	−0.75 (0.77)^A,C,D^	0.28 (0.54)^B^	0.24 (0.50)^B^	<0.001
Attention	0.15 (0.72)^B,C^	−0.65 (0.74)^A,C,D^	−0.29 (0.63)^A,B,D^	0.06 (0.57)^B,C^	<0.001
NPI total score*	9 (4, 17)^C,D^	8 (2, 14)^C,D^	4 (0, 9)^A,B,D^	18 (11, 30)^A,B,C^	<0.001

Abbreviations: AD = Alzheimer disease; bvAD = behavioral variant AD; CDR = clinical dementia rating; lvPPA = logopenic variant primary progressive aphasia; MMSE = Mini-Mental State Examination; NPI = neuropsychiatric inventory; PCA = posterior cortical atrophy.

Categorical variables are presented as n (%), continuous variables are presented as mean (SD), and variables indicated with an asterisk (*) are presented as median (interquartile range). *p* Values provided for the overall χ² tests (categorical variables), one-way ANOVA tests (continuous variables), or Kruskal-Wallis tests (continuous variables indicated with an asterisk) followed by pairwise χ² tests (Bonferroni correction for multiple testing), Tukey HSD tests, and Wilcoxon rank tests (Bonferroni correction for multiple testing) when applicable. Significant (*p* < 0.05) pairwise comparisons are indicated relative to the typical AD (^A^), PCA (^B^), lvPPA (^C^), and bvAD (^D^) groups. Missing data are reported in eTable 1.

### Statistical Analyses

Statistical analyses were performed using R studio version 4.3.2. Participant characteristics were compared using the χ² test (categorical variables), one-way analysis of variance (continuous variables), or the Kruskal-Wallis test (skewed continuous variables). Significant group differences were further assessed using pairwise χ² or Fisher exact tests with Bonferroni correction for multiple testing, Tukey honestly significant difference tests, and Wilcoxon rank tests with Bonferroni correction for multiple testing, respectively.

For our survival analyses, raw median survival estimates were first obtained from Kaplan-Meier analysis for survival from the first visit to the event (i.e., death or censoring) for typical AD; for atypical AD combined; and for PCA, lvPPA, and bvAD separately. Of note, because 48% of the bvAD sample had deceased, the median survival estimate presented for bvAD is an extrapolation. Statistical difference between the survival curves was determined based on (pairwise) log-rank tests. Second, to further investigate the association of atypical AD (PCA, lvPPA, and bvAD) with mortality beyond other potential mortality risk determinants, Cox proportional hazard models were constructed including sex, education, MMSE score, *APOE*ε4 carriership (noncarriers, heterozygote carriers, and homozygote carriers), and age at the first visit (model 1), followed by addition of atypical AD as a whole (model 2) or as separate variants (model 3), while using typical AD as the reference group. A likelihood ratio test was performed for models 1 and 2 to determine whether including atypical AD as a predictor provides a significantly improved model fit. We tested all possible interactions for the specific atypical AD diagnoses (i.e., atypical AD* *APOE*ε4 carriership, age, or sex) using Cox proportional hazard models including the same set of covariates where applicable. Of note, fewer patients with typical AD (n = 1,665) and atypical AD (n = 266; PCA [n = 106], lvPPA [n = 80], and bvAD [n = 80]) were included in the covariate-adjusted models because of missing data (eTable 1). Sensitivity analyses were performed for a subgroup of patients in the dementia stage. Furthermore, to test the robustness of our findings for implemented methodology, we further performed a restricted median survival time (RMST) analysis for the typical AD group and atypical AD variants. The RMST restriction time was defined as the shortest maximum follow-up across the diagnostic groups, and the model was adjusted for the same set of covariates.

### Standard Protocol Approvals, Registrations, and Patient Consents

The study protocol of the ADC was approved by the ethical review board of the VU University Medical Center (2016.061). Written informed consent was obtained from all patients for the use of their data for research purposes.

### Data Availability

Data can be made available on reasonable request.

## Results

### Participant Characteristics

As shown in Figure 1, the initial sample included 2,741 amyloid-positive patients who visited the memory clinic between February 1998 and November 2023. Participants were excluded if they had an unknown date of death (n = 2), a known autosomal dominant genetic variant for AD (n = 14), a CBS diagnosis (n = 18), an isolated and severe amnestic syndrome diagnosis (n = 56), a different PPA diagnosis than lvPPA (n = 27), or a syndromic diagnosis of SCD at the first visit (n = 35). In the remaining sample (n = 2,589), 95 patients with PCA, 52 patients with lvPPA, and 51 patients with bvAD were already classified based on multidisciplinary consensus and previous research studies.^30,39-41^ During retrospective case finding, 157 patients were flagged as potentially atypical. After more detailed inspection, 82 patients (17 PCA, 34 lvPPA, and 30 bvAD) were included in the atypical AD group while the remaining 75 patients were excluded. The typical AD group was further defined by excluding those with a non-AD diagnosis at the first visit (n = 68) or at the last visit (n = 60) and those with an MCI diagnosis at the *last* visit (n = 305).

The final sample included 2,081 amyloid-positive patients grouped into typical AD (n = 1,801) and atypical AD (n = 280; PCA [n = 112], lvPPA [n = 86], and bvAD [n = 82]). The Table presents the characteristics around the time of the first visit per diagnostic group. Overall, the sample included 1,004 women (52%), and the mean age was 65 ± 8 years. After a mean follow-up time of 5.4 ± 3.4 years (range 0–18 years), 1,304 (63%) of the patients had died. eTable 2 presents the sample characteristics after excluding patients with missing data (i.e., the sample for covariate-adjusted analyses).

### Estimated Survival Durations for Atypical AD

**Figure 2:**
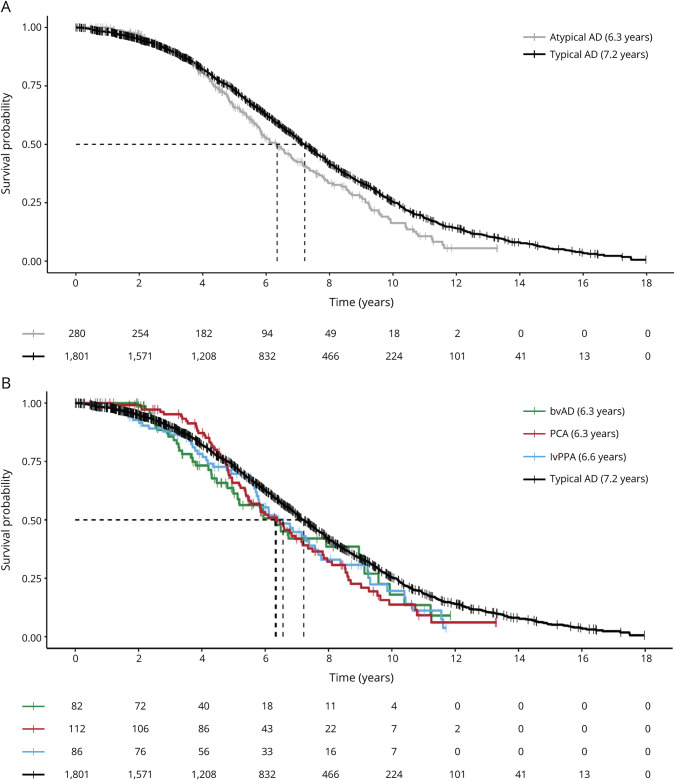
Kaplan-Meier Curves for Typical AD and Atypical AD Kaplan-Meier curves show the survival curves for all atypical AD variants combined (A) and for the separate atypical AD variants (B). Vertical lines indicate the median survival durations as reported in the legend. Of note, <50% of the bvAD had deceased (i.e., 48%). AD = Alzheimer disease; bvAD = behavioral variant AD; lvPPA = logopenic variant primary progressive aphasia; PCA = posterior cortical atrophy.

The estimated median survival time for atypical AD was 6.3 years (95% CI 5.8–6.9), which was significantly shorter than the median survival time of 7.2 years (95% CI 7.0–7.5, *p* = 0.02) for typical AD (Figure 2A). Across separate diagnostic groups, survival durations were consistently shorter compared with typical AD but these did not reach significance for these smaller subgroups, that is, 6.3 years for PCA (95% CI 5.5–7.3; *p* = 0.055), 6.3 years for bvAD (95% CI 5.0–9.1; *p* = 0.121), and 6.6 years for lvPPA (95% CI 5.7–7.7; *p* = 0.110) (Figure 2B).

### Associations Between Specific Atypical AD Variants and Mortality

**Figure 3:**
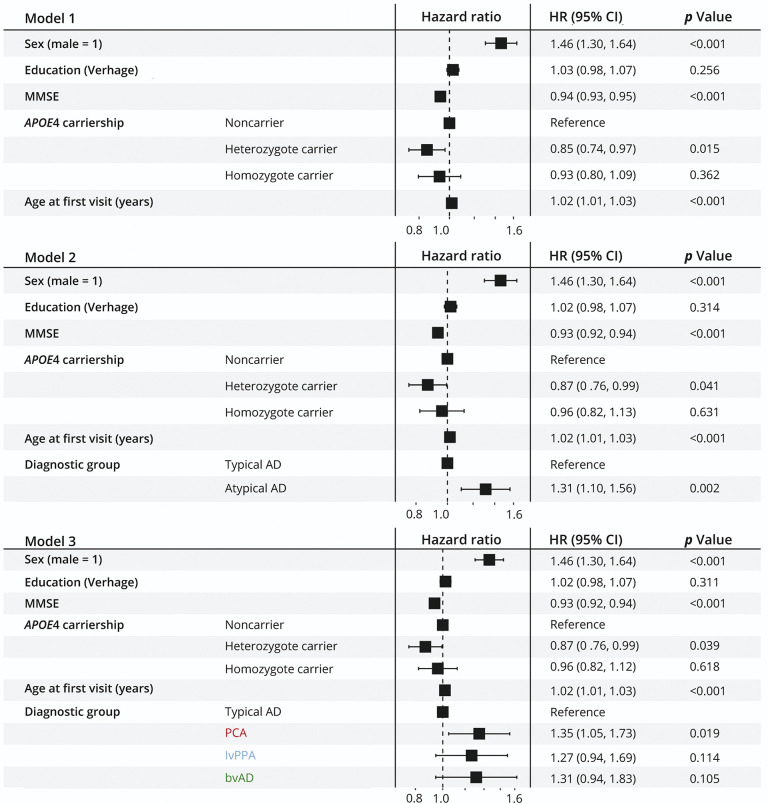
Cox Proportional Hazard Models to Evaluate the Influence of Atypical AD Variants (PCA, lvPPA, and bvAD) on Mortality Cox proportional hazard models (n = 1931, number of events = 1,195) were constructed to evaluate the impact of an atypical AD diagnosis on mortality while also exploring the associations for age, sex, education, MMSE score, and *APOE*ε4 genotype. AD = Alzheimer disease; bvAD = behavioral variant AD; HR = hazard ratio; lvPPA = logopenic variant primary progressive aphasia; MMSE = Mini-Mental State Examination; PCA = posterior cortical atrophy.

Cox proportional hazard models (Figure 3, model 1) showed that male sex (hazard ratio [HR] = 1.46 [95% CI 1.30–1.64], *p* < 0.001), a lower MMSE score (HR = 0.94 [0.93–0.95] per Mini-Mental State Examination [MMSE] point, *p* < 0.001), and older age (HR = 1.02 [1.01–1.02] per year, *p* < 0.001) were associated with increased mortality risk. Conversely, heterozygous *APOE*ε4 carriership was associated with decreased mortality risk (HR = 0.85 [0.74–0.97], *p* = 0.015) relative to noncarriers. There were no associations with mortality for homozygous *APOE*ε4 carriership (HR = 0.93 [0.80–1.09], *p* = 0.362) vs noncarriers, nor for education (HR = 1.03 [0.98–1.07], *p* = 0.256).

While simultaneously modeling mortality risk determinants, the fit of the model improved when atypical AD was included (χ^2^ = 8.88, df = 1, *p* = 0.003) according to the likelihood ratio test. In this model (Figure 3, model 2), patients with atypical AD remained at 31% higher risk of mortality than patients with typical AD (HR = 1.31 [1.10–1.56], *p* = 0.002). Within the atypical AD group, associations for separate diagnostic groups (Figure 3, model 3) were significant for PCA (HR = 1.35 [1.05–1.73]; *p* = 0.0019) and similar in direction (but not significant) for lvPPA (HR = 1.27 [0.84–1.69]; *p* = 0.114) and bvAD (HR = 1.31 [0.94–1.83]; *p* = 0.105). There were no interactions for atypical AD with *APOE*ε4 status, age, and sex (eTable 3).

### Sensitivity Analyses

Sample characteristics of the dementia-only subgroup are provided in eTable 4. The unadjusted median survival estimates were not significantly different for atypical AD (6.1 years [95% CI 5.8–6.9]) and typical AD (6.8 years [95% CI 6.6–7.0], *p* = 0.1; eFigure 1). However when adjusting for age, sex, education, MMSE score, and *APOE*ε4 status, patients with atypical AD remained at 24% higher risk of mortality (HR = 1.24 [1.04–1.48]; *p* = 0.019) and including atypical AD as a predictor significantly improved the model fit (χ^2^ = 5.24, df = 1, *p* = 0.022) according to the likelihood ratio test (eFigure 2). Across the smaller subgroups, associations with mortality remained consistent in direction, but no longer reached significance for PCA (HR = 1.26 [0.98–1.61]; *p* = 0.075).

To further test the robustness of our findings for implemented methodology, we further performed a RMST analysis for the typical AD group and atypical AD variants. At a restriction time of 13.3 years, the adjusted RMST for the combined atypical AD phenotypes was 6.2 years, which was significantly shorter than the RMST for typical AD (6.9 years; RMST difference = −0.702; 95% CI −1.299 to −0.105; *p* = 0.021). These results are similar to those of the Cox proportional hazard model.

## Discussion

In this study, we estimated survival probabilities for patients with AD biomarker–confirmed typical AD vs atypical AD (PCA, lvPPA, and bvAD only) and evaluated the effect of these atypical AD diagnoses while taking into account other risk determinants (sex, education, disease stage, *APOE*ε*4* carriership, and age). We observed that the estimated median survival time was almost 1 year shorter in patients with atypical AD (6.3 years) compared with the typical AD cohort (7.2 years). Across the PCA, lvPPA, and bvAD diagnostic subgroups, survival estimates were consistently shorter than for typical AD (6.3, 6.6, and 6.3 years, respectively), although these differences were not statistically significant. The atypical AD variants combined (i.e., a PCA, lvPPA, or bvAD diagnosis) conferred a 31% relative risk increase for mortality compared with typical AD, after adjustment for other determinants of mortality. Taken together, patients with an atypical AD phenotype have a shorter life expectancy compared with those with a typical phenotype.

Because survival durations depend on more than just phenotypic variations, we also assessed known mortality modifiers. We observed increased mortality risk for male sex, a lower MMSE score, *APOE*ε4 noncarriers, and patients who were older at the time of their first visit. Although higher mortality risk for atypical AD compared with typical AD was partly explained by these factors, we detected a significant independent effect of an atypical AD diagnosis on mortality risk. This raises the question which other factors, related to atypicality, may contribute to the observed difference. From a pathophysiologic perspective, phenotypic heterogeneity in PCA, lvPPA, and bvAD corresponds to variant-specific patterns of pathology in the brain,^3,42,43^ highlighting potential differences in vulnerability of specific brain regions. Atypical variants are mostly associated with a neocortical predominant pattern of pathology and atrophy, which has been associated with faster disease progression,^44^ although the mechanisms underlying this phenomenon are unknown. From a clinical perspective, alternative (or additional) explanations for faster progression could be related to consequences of under-recognition, diagnostic delays, and limited tailored patient support or management.^4,45^ The latter could result from resources being tailored to challenges encountered by (often older) patients who have typical AD rather than (often younger) patients who have atypical AD. Future research into the pathophysiologic and clinical factors contributing to increased mortality in atypical AD may provide valuable insight into modifiable factors that could reduce the currently observed survival differences.

Faster clinical progression has been previously observed in patients with a neuropathologically confirmed AD diagnosis presenting with initial predominant nonamnestic symptoms^6^ and in individuals from a nonmemory cluster (mostly executive and some visuospatial function^46^) vs a memory cluster.^5^ The latter study reported increased mortality risk but excluded atypical AD variants in 2 cohorts and did not specify clinical AD variants. This study extends findings from atypical AD phenotypes to consensus-based atypical AD variants, for which survival estimates have been relatively limited. For PCA, the most common atypical variant of AD, previous studies reported a median survival time of 8 years from study enrollment (n = 12)^27^ and 10.3 years from symptom onset (n = 65).^28^ The first study reported longer survival durations which may be explained by their operationalization of survival and inclusion of young-onset patients (56 ± 4.0 years of age) who still scored relatively high on the MMSE (23.6 ± 3.6). The second study reported fairly similar survival durations considering our observed median survival estimate of 6.3 years and median concern duration of 3.0 years. Of note, although PCA is highly specific for underlying AD,^31^ both studies lacked complete biomarker confirmation (i.e., 6/12 and 8/65 individuals with confirmed amyloid, respectively). For lvPPA, our median survival estimate is relatively long compared with previous estimates of 6 years from symptom onset and 5 years from diagnosis (n = 35).^29^ However, the previous study included a slightly older sample (72 ± 10 years of age at diagnosis), and although lvPPA is also highly specific to AD,^30^ presence of underlying AD pathology was not confirmed. Hence, effects of older age on mortality and possible but unconfirmed differences in underlying pathology could have resulted in slightly different estimates. For bvAD, a relatively recently defined phenotype,^3^ our estimate of 6.3 years cannot be compared with previous literature and should be interpreted with caution because fewer than 50% (48%) of this group had deceased.

Of interest, our observed median survival of 7.2 years for typical AD is higher than a previous estimate of 6.2 years derived from a sample from the same memory clinic.^15^ It is important to note that this previous study included only patients with dementia (i.e., no MCI) and did not separately evaluate patients with an atypical variant, who seem to have shorter survival durations. In this study, patients with typical AD were largely comparable with patients with atypical AD regarding age, concern duration, and disease severity. When assessed separately, only individuals diagnosed with PCA were relatively younger at their first visit compared with the typical AD cohort. Of interest, although the raw median survival estimates in the PCA, lvPPA, and bvAD diagnostic groups were consistently lower than in typical AD, this difference was only significant for PCA. This finding may be due to the relatively large sample size for PCA compared with lvPPA and bvAD. Alternatively, the relatively young age at onset of PCA might result in a more progressive disease course as previously reported for early-onset AD (EOAD).^7-9,14^

Comparisons between previous survival studies highlight the importance of considering survival definitions and sample compositions. This study defined survival from the first visit rather than diagnosis to avoid bias from diagnostic delays (although the time between these was negligible across diagnostic groups). Regarding sample composition, we included patients with biomarker-confirmed typical and atypical AD with a diagnosis of MCI or dementia. MCI and dementia were combined in our main analyses because defining these stages is complex for atypical phenotypes. For example, scales such as the clinical dementia rating rely strongly on memory and orientation, domains primarily affected in typical amnestic-predominant AD. Furthermore, for patients in the same stage of disease, interference in daily life could differ for patients with predominant atypical visual, language, or behavioral concerns vs those with more typical memory and orientation domains. Based on this, we included all patients with AD regardless of their syndrome diagnosis in both the typical and atypical diagnostic groups and included the MMSE score in all models as a proxy of disease stage. Furthermore, we performed a sensitivity analysis among only the patients with dementia in whom characteristics for the atypical AD groups remained similar, whereas patients in the typical AD group were older, more often had a clinical dementia rating ≥1.0, and performed worse on the MMSE. When taking age, sex, education, MMSE score, and *APOE*ε4 status into account, an atypical AD diagnosis seemed a risk factor of mortality in the full sample (31%) and in the dementia-only sample (24%).

Of note, both a younger age and *APOE*ε4 carriership did not contribute to increased mortality risk and, perhaps surprisingly, seemed to be somewhat protective in this study. Regarding age, atypical AD variants are more often early onset and previous studies have reported that younger age in patients with EOAD may drive a relatively progressive disease course compared with late-onset AD (LOAD).^7-9,25,26^ However, studies have reported conflicting results^24^ with shorter,^14^ similar,^15^ or even longer^9^ survival durations in EOAD vs LOAD cohorts. In this study, younger age was not independently related to increased mortality risk, and no significant interaction between the specific atypical diagnoses and age was observed. It is an intriguing possibility that atypical AD variants might have partly driven previously reported associations between EOAD and shorter survival durations, although only around one-third of patients with EOAD present with atypical phenotypes.^35,47^ Regarding the effect of *APOE*ε4 carriership, the observed protective effect of *APOE*ε4 heterozygosity compared with noncarriership may be counterintuitive, given the strong and dose-dependent effect of *APOE*ε*4* on AD pathology, symptom onset, and possibly mortality.^12^ However, other studies have reported that *APOE*ε4 carriership can be associated with a *later* disease onset in EOAD.^48^ One explanation could be the relatively strong association of *APOE*ε4 with medial temporal lobe pathology and memory decline, which are both characteristic features for typical, amnestic LOAD and relatively spared in EOAD and atypical AD.^25,49^ Indeed, in the sample of patients with PCA and lvPPA, *APOE*ε4 carriership related to more initial medial temporal lobe atrophy, but rates of medial temporal atrophy and tau accumulation were slower and cognitive associations were relatively weak.^50^ Regarding mortality, associations with *APOE*ε*4* status may be complex because of pleiotropic associations across life stages and both beneficial and detrimental effects across different diseases.^13^ Future studies are required to assess the mechanisms underlying differential mortality risk associated with *APOE*ε4 in typical vs atypical AD.

A strength of this study is the relatively large sample size and long follow-up time for typical and atypical patients with a biomarker-confirmed AD diagnosis. The relatively young age of patients with typical AD provides a representative comparison with the atypical AD groups. Furthermore, we aimed to limit potential under-recognition or misclassification of patients with atypical AD (1) by including both MCI and AD dementia diagnoses because the distinction between the two is based on amnestic and subsequent multidomain cognitive impairment and (2) by performing a retrospective case-finding procedure, which was particularly relevant for patients who visited the memory clinic before the current consensus criteria for atypical AD were published.^2,3,36-38^ This study also has several limitations. First, the raw survival estimate for bvAD warrants caution because 48% of the patients in this group were deceased. Second, patients were selected from a tertiary memory clinic cohort and may have entered the clinic in different disease stages, for example, after a clinical workup elsewhere. Our correction for disease stage based on the MMSE score is suboptimal for atypical AD, given its strong reliance on the typical memory and orientation domains. Third, effects of co-pathologies, comorbidities, and causes of death were not evaluated in this study. Although all patients were amyloid-positive, this does not exclude the presence and detrimental effects of other pathologies. Furthermore, although the extent of comorbidity is expected to be relatively equal across groups, given the comparable group characteristics, there could have been differences in causes of death between groups due to comorbidities or due to the specific symptomatology (e.g., a fall or accident in case of visuospatial problems). However, documentation on causes of death was too sparse to evaluate these factors in this study. Fourth, “atypical AD” as defined in this study is restricted to PCA, lvPPA, and PCA, which limits generalizability of these results to other proposed variants (e.g., dysexecutive AD, CBS, or other language presentations with AD as underlying etiology that do not fulfill lvPPA criteria). Finally, missing data reduced the sample size in the covariate-adjusted analyses compared with the raw survival analyses. Based on similar group characteristics and the comparability in outcomes of raw and adjusted analyses, the impact of this limitation is expected to be limited.

In conclusion, survival durations for the atypical AD variants included in this study are shorter than for typical AD; thus PCA, lvPPA, and bvAD diagnoses seem to predispose for increased risk of mortality beyond age, sex, education, *APOE*ε4 carriership, and disease severity. Findings were numerically consistent across the smaller PCA, lvPPA, and bvAD diagnostic subgroups, although only reaching statistical significance for PCA. These findings emphasize the importance of recognizing clinical heterogeneity among patients with AD. Future studies should explore the extent to which these results generalize to atypical AD as a whole and can leverage our findings to explore (potentially modifiable) risk factors of atypical AD and intervention strategies that can increase survival time, such as preventing diagnostic delays and providing tailored care.

## Glossary

AD: Alzheimer disease
ADC: Amsterdam Dementia Cohort
bvAD: behavioral AD
CBS: corticobasal syndrome
EOAD: early-onset AD
LOAD: late-onset AD
lvPPA: logopenic variant primary progressive aphasia
MCI: mild cognitive impairment
MMSE: Mini-Mental State Examination
PCA: posterior cortical atrophy
RMST: restricted median survival time
SCD: subjective cognitive decline
TMT: Trail Making Test
VAT: Visual Association Test
